# Quality of prenatal care questionnaire: psychometric testing in an Australia population

**DOI:** 10.1186/s12884-015-0644-7

**Published:** 2015-09-10

**Authors:** Wendy Sword, Maureen Heaman, Mary Anne Biro, Caroline Homer, Jane Yelland, Noori Akhtar-Danesh, Amanda Bradford-Janke

**Affiliations:** School of Nursing, Faculty of Health Sciences, University of Ottawa, 451 Smyth Road, Ottawa, ON K1H 8M5 Canada; College of Nursing and Departments of Community Health Sciences and Obstetrics, Gynecology and Reproductive Sciences, College of Medicine, Faculty of Health Sciences, University of Manitoba, 89 Curry Place, Winnipeg, MB R3T 2N2 Canada; School of Nursing and Midwifery, Faculty of Medicine, Nursing and Health Sciences, Monash University, Frankston, VIC 3800 Australia; Centre for Midwifery, Child and Family Health, Faculty of Health, University of Technology Sydney, 15 Broadway, Ultimo, NSW 2007 Australia; Healthy Mothers Healthy Families Research Group, Murdoch Childrens Research Institute and General Practice and Primary Health Care Academic Centre, University of Melbourne, Parkville, 3052 Australia; School of Nursing and Department of Clinical Epidemiology & Biostatics, Faculty of Health Sciences, McMaster University, 1280 Main Street West, Hamilton, ON L8S 4K1 Canada; Gilbrea Centre for Studies in Aging, Faculty of Social Sciences, McMaster University, 1280 Main Street West, Hamilton, ON L8S 4M4 Canada

## Abstract

**Background:**

The quality of antenatal care is recognized as critical to the effectiveness of care in optimizing maternal and child health outcomes. However, research has been hindered by the lack of a theoretically-grounded and psychometrically sound instrument to assess the quality of antenatal care. In response to this need, the 46-item Quality of Prenatal Care Questionnaire (QPCQ) was developed and tested in a Canadian context. The objective of this study was to validate the QPCQ and to establish its internal consistency reliability in an Australian population.

**Methods:**

Study participants were recruited from two public maternity services in two Australian states: Monash Health, Victoria and Wollongong Hospital, New South Wales. Women were eligible to participate if they had given birth to a single live infant, were 18 years or older, had at least three antenatal visits during the pregnancy, and could speak, read and write English. Study questionnaires were completed in hospital. A confirmatory factor analysis (CFA) was conducted. Construct validity, including convergent validity, was further assessed against existing questionnaires: the Patient Expectations and Satisfaction with Prenatal Care (PESPC) and the Prenatal Interpersonal Processes of Care (PIPC). Internal consistency reliability of the QPCQ and each of its six subscales was assessed using Cronbach’s alpha.

**Results:**

Two hundred and ninety-nine women participated in the study. CFA verified and confirmed the six factors (subscales) of the QPCQ. A hypothesis-testing approach and an assessment of convergent validity further supported construct validity of the instrument. The QPCQ had acceptable internal consistency reliability (Cronbach’s alpha = 0.97), as did each of the six factors (Cronbach’s alpha = 0.74 to 0.95).

**Conclusions:**

The QPCQ is a valid and reliable self-report measure of antenatal care quality. This instrument fills a scientific gap and can be used in research to examine relationships between the quality of antenatal care and outcomes of interest, and to examine variations in antenatal care quality. It also will be useful in quality assurance and improvement initiatives.

## Background

Quality of care has received much attention in recent discourses on health care systems and patient outcomes. The Institute of Medicine [1] defined quality of health care as “the degree to which health services for individuals and populations increase the likelihood of desired health outcomes and are consistent with current professional knowledge” (p. 232). According to Donabedian [2, 3], quality of care can be broadly conceptualized and operationalized according to three main attributes: structure (the setting in which care occurs); process (what is done in the giving and receiving of care); and outcomes (the effect on health status). Campbell, Roland, and Buetwo [4] adapted Donabeidan’s [2, 3] model and suggested two key dimensions of quality of care, access and effectiveness; access is defined as encompassing geographic or physical, affordability, and availability whereas effectiveness includes clinical or technical effectiveness and the effectiveness of interpersonal interactions. They considered outcomes a consequence of care rather than a component of care [4].

The literature on quality in antenatal care addresses both the structural and process elements in Donabedian’s model. A commonly identified structural element is access to services, which includes availability, geographic access, ease of scheduling appointments, hours of service delivery, telephone availability of care provider to address pressing questions or concerns, and access to educational materials [5–8]. Other aspects of structure that are viewed as impacting quality of care are wait times [5, 6], the physical setting (e.g., cleanliness, ventilation, privacy) [5, 6, 8, 9], and staff and care provider characteristics (e.g., clinical knowledge and skills, efficiency) [5–9].

Clinical care processes that have been identified as components of quality antenatal care encompass: assessment, screening, and monitoring [6–10]; information exchange, health promotion, teaching, and counselling [5–9, 11–13]; woman-centred care, shared decision making, and self-care [6–8, 11, 13]; continuity of care [5–8]; normalization of pregnancy and promotion of normal processes [7, 8]; and adherence to evidence-based clinical practice guidelines [14, 15]. Interpersonal care processes that contribute to quality antenatal include: listening carefully and understanding [7, 8, 12]; showing respect [5–9, 12, 13]; adequate time with care provider [6, 8, 9, 12]; approachable interpersonal style [8, 13]; emotional support [8, 13]; and cultural sensitivity and competence [6–8].

Few studies have explicitly examined relationships between quality of antenatal care and outcomes for women and infants. However, available evidence suggests that providing women with quality antenatal care is important in mitigating poor outcomes. In a randomized controlled trial (RCT), Klerman et al. [16] assessed the impact of quality antenatal care, defined as additional visits with psychosocial support, on maternal (smoking cessation, weight gain, perceived mastery) and infant (gestational age, birth weight) outcomes. Although preterm birth, self-reported smoking cessation, and caesarean birth rates were improved in the care group with additional visits, small numbers of women and infants having these adverse outcomes precluded the detection of statistically significant differences. Ricketts et al. [17] similarly found that providing enhanced services targeting risk factors, such as smoking and psychosocial problems, was effective in resolving risks and, subsequently, in reducing the risk of low birth weight. In a RCT of an early antenatal health promotion workshop designed to supplement usual care, workshop attendance resulted in significant improvements in health behaviours, including diet and physical activity [18].

Other studies have focused on adherence to recommended antenatal care guidelines or content as a quality indicator. White et al. [19] examined relationships between adherence to selected recommendations for antenatal care published by the Society of Obstetricians and Gynaecologists of Canada and preterm birth. They found no association between the content of antenatal care and preterm birth. In contrast, Handler et al. [20] reported that lower adherence to recommended antenatal care content was significantly associated with low birth weight and preterm birth among women receiving antenatal care at physicians’ offices. Vonderheid et al. [21] found a positive association between the number of health promotion topics discussed and health behaviours during pregnancy.

The introduction of group antenatal care, and specifically CenteringPregnancy, was a strategy to improve quality of care and perinatal outcomes by addressing the recommended content for optimizing antenatal care [22]. Group antenatal care allows for more time with health care professionals compared to traditional individual appointments, thus enhancing educational opportunities, and participants can benefit from the social support that is acquired through interaction and linkages with other women [22, 23]. A Cochrane review of group versus conventional antenatal care for women included two RCTs of CenteringPregnancy, one a multi-site, three-arm RCT that integrated skills-building in HIV STD prevention with group antenatal care [24], and the other a two-arm RCT conducted in two military settings [25]. No statistically significant differences were found between women who received group versus standard antenatal care on the primary outcomes (gestational age at birth, low birth weight, small-for-gestational age, and perinatal mortality); however, women who participated in group antenatal care had higher levels of antenatal knowledge, readiness for labour and birth, and satisfaction with care [23]. In the three arm trial, women who received group antenatal care plus skills-building versus standard care were less likely to have a repeat pregnancy at 6 months and had more condom use, less unprotected sex, and greater communication about safe sex [23, 26].

Although published studies suggest the importance of antenatal care quality, research in this area has been hindered by the lack of a robust instrument that comprehensively measures quality of antenatal care. As stated by Alexander and Kotelchuck [27], “…the rigorous scientific evidence of its [antenatal care] effects on health outcomes, health-related behaviors, health care utilization, and health care costs is meager and insufficient” (p. 314). They note that research on the measurement and impact of quality of antenatal is one key area for further research. In response to this need, our research team developed and tested the Quality of Prenatal Care Questionnaire (QPCQ) in a Canadian context.

The QPCQ is a 46-item instrument with six validated subscales: Information Sharing, Anticipatory Guidance, Sufficient Time, Approachability, Availability, and Support and Respect [28]. Construct validity was further demonstrated using a hypothesis testing approach; there was a significant positive association between women’s ratings of the quality of antenatal care and their satisfaction with care (*r* = 0.81) [28]. Convergent validity, another approach to construct validity, was demonstrated by a significant positive correlation (*r* = 0.63) between the “Support and Respect” subscale of the QPCQ and the “Respectfulness/Emotional Support” subscale of the Prenatal Interpersonal Processes of Care instrument (PIPC) [13, 28]. The overall QPCQ has acceptable internal consistency reliability (Cronbach’s alpha = 0.96), as does each of the subscales (Cronbach’s alpha = 0.73 to 0.93) [28]. The test-retest reliability result (Intra-class correlation coefficient = 0.88) indicated stability of the instrument on repeat administration approximately one week later; temporal stability testing confirmed that women’s ratings of their quality of antenatal care did not change as a result of giving birth or between the early postpartum period and 4 to 6 weeks postpartum [28].

Given the universality of antenatal care in developed countries, we were interested in testing the QPCQ in other settings. The objective of this study therefore was to confirm the factor structure of each of its six subscales, further evaluate construct validity, and establish the internal consistency reliability of the QPCQ in an Australian population.

## Methods

### Setting and sample

In Australia, public antenatal care is offered to women and their families through public hospital antenatal clinics where women often see different midwives and/or doctors at each pregnancy visit. Alternatively, women attend community-based practitioners (usually general practitioners) or access care via shared care arrangements where care is shared between a community-based general practitioner and hospital antenatal clinic. Approximately 70 % of antenatal care is offered through the public system and funded through public insurance, which is accessible to all (Medicare). Private care options include an obstetrician, general practitioner-obstetrician or independent midwife as the care provider.

Study participants were recruited from two public maternity services in two Australian states: Victoria and New South Wales. These two large maternity services were Monash Health in the southeastern suburbs of the city of Melbourne, Victoria, which provides maternity care for approximately 9000 women per year within three hospitals (Monash Medical Centre, Dandenong Hospital, and Casey Hospital) and Wollongong Hospital in a non-metropolitan area of New South Wales, which provides care for approximately 2200 women per year.

Women were eligible to participate if they had given birth to a single live infant, were 18 years or older, had at least three antenatal visits during the pregnancy, and could speak, read and write English. We excluded women who had an intellectual disability or mental illness that precluded giving informed consent and women who had experienced a seriously ill infant or perinatal death. We aimed to recruit a sample of 300 women. A sample size of 300 was determined to be sufficient as Devellis [29] suggests that a sample size of 200 is adequate in most cases of factor analysis and Comrey and Lee [30] state that a sample size of 300 is acceptable to calculate Cronbach’s alpha in this context.

### Recruitment and data collection procedures

We used a convenience sampling approach. In both states, a research assistant visited the postnatal wards daily and made contact with the midwife in charge to ascertain eligibility of women. If a woman met the eligibility criteria the research assistant checked to see whether it was appropriate to visit her at that time. The research assistant then visited and invited women to participate by providing them with a verbal explanation and written information about the study. Signed, informed consent was obtained from those who agreed to participate. Study participants then completed the QPCQ, the Patient Expectations and Satisfaction with Prenatal Care (PESPC) instrument [31], the PIPC instrument [13], and a brief sociodemographic information form. For the purposes of this study, we changed the term “prenatal” to “antenatal” in the QPCQ questionnaire items to reflect well-accepted and familiar terminology in Australia, which is based on British English rather than North American English. Each participant received a $20 gift voucher in appreciation for her time and contribution to the study.

The PESPC and the PIPC were administered to enable us to use different approaches to construct validity testing of the QPCQ. The PESPC is a valid and reliable 41-item self-administered questionnaire designed to measure pregnant women’s expectations and satisfaction with their antenatal care. The Satisfaction subscale used in our analysis has acceptable internal consistency (Cronbah’s alpha = .94) [31]. The 30-item PIPC has seven scales that reflect three underlying dimensions: Communication, Patient-Centered Decision Making, and Interpersonal Style. The seven scales have acceptable internal consistency reliability (Cronbach’s alpha = 0.66 to 0.85) and the PIPC has acceptable construct validity [13]. This instrument was chosen as there is no other measure of quality of antenatal care and the PIPC is a measure of the quality of one dimension of care described by Campbell et al. [4], interpersonal processes of care.

Data collection took place between November 2012 and January 2013 at Monash Health and between March 2013 and July 2013 at Wollongong Hospital. Ethics approval was obtained from the Hamilton Health Sciences/McMaster University Faculty of Health Sciences Research Ethics Board, Monash Health Human Research and Ethics Committee, Monash University Human Research Ethics Committee, and the University of Wollongong and Illawarra Local Health District Human Research Ethics Committee.

### Data analysis

Descriptive statistics were used to summarize the sociodemographic characteristics of study participants and to determine subscale means and standard deviations. Each subscale mean score was calculated by first reversing the scores of reverse-scored items in the subscale, then summing the scores for the subscale items and dividing the sum by the number of items. Confirmatory factor analysis (CFA) was used to confirm the factor structure of the underlying dimensions of the construct that were previously identified in the initial psychometric testing of the QPCQ [28]. We assessed the items loading on each factor (subscale), that is, the correlations between each item and the subscale to which it belongs [32]. Additionally, we determined the standardized regression estimates (factor loadings) between each factor and the loaded items on that factor. A hypothesis testing approach was used to further assess construct validity [32]. We hypothesized that women who rated the quality of their antenatal care higher would have higher ratings of satisfaction with antenatal care using the PESPC. The Pearson correlation between the total QPCQ score and the Satisfaction subscale score of the PESPC instrument was estimated. To assess convergent validity, a type of construct validity, the Pearson correlation between PIPC subscale scores and scores on QPCQ subscales that captured the same constructs was estimated [32]. Internal consistency reliability of the QPCQ and each of its six subscales was assessed using Cronbach’s alpha [32]. The *root mean square error of approximation* (RMSEA) statistic was used to assess the goodness-of-fit of the CFA model. The RMSEA is the most commonly used index [33] for the evaluation of CFA and estimates the lack of fit of the model. A value of RMSEA ≤ 0.05 indicates a close fit, a value between 0.05 and 0.10 suggests a reasonable fit, and a value larger than 0.10 is indicative of a poor model [34, 35]. CFA was conducted using Amos 22 statistical program. All other statistical analyses were performed using SPSS version 18.0 or the Stata SE/12.1 program.

## Results

A total of 299 women were recruited into the study, 150 from Monash Health and 149 from Wollongong Hospital. Their sociodemographic characteristics are presented in Table 1. There was variation across all categories, and it was expected that the majority of women would report English as the language spoken most frequently at home given that ability to communicate in English was an eligibility criterion. The QPCQ factor (subscale) means and standard deviations are presented in Table 2. The mean scores for the factors ranged from 3.84 to 4.27 out of a total score of 5. The factor “Information Sharing” had the highest mean rating while “Anticipatory Guidance” had the lowest mean rating. CFA verified and confirmed the presence of the six factors. Table 3 presents the list of items on each factor, corrected item-total subscale correlations, Cronbach’s alpha if the item is deleted from the subscale, and factor loadings. The overall QPCQ had acceptable internal consistency reliability (Cronbach’s alpha = 0.97) as did each of the factors (subscales) (Cronbach’s alpha = 0.74 to 0.95) (see Table 3).

**Table 1:** Participant Characteristics (*N* = 299)

Characteristic	Mean (SD)^a^
Maternal age (years) (*n* = 298)	30.3 (5.7)
Gestational age at first antenatal care visit (weeks) (*n* = 292)	12.9 (6.2)
Gestational age at delivery (weeks) (*n* = 297)	39.2 (1.4)
Infant birth weight (grams) (*n* = 296)	3387.3 (507.3)
	*n* (%)^a^
Marital status (*n* = 296)	
Married	172 (58.1)
Living with a partner	88 (29.7)
Single (never married)	32 (10.8)
Divorced	4 (1.4)
Household income (*n* = 261)	
No income	2 (0.8)
Under $20,000	31 (11.9)
$20,001 to $50,000	60 (23.0)
$50,001 to $80,000	61 (23.4)
$80,001 to $100,000	55 (21.1)
$100,001 and over	52 (19.9)
Highest level of education (*n* = 299)	
Less than high school	43 (14.3)
Completed high school	60 (20.1)
Some community college or TAFE	14 (4.7)
Completed community college or TAFE	64 (21.4)
Some university	24 (8.0)
Completed bachelor’s degree	56 (18.7)
Post graduate degree	38 (12.7)
Born in Australia (*n* = 299)	
Yes	196 (65.6)
No	103 (34.4)
Language spoken most often at home (*n* = 291)	
English	236 (81.1)
Other	55 (18.9)
Antenatal care provider^b^ (*n* = 299)	
Midwife	201 (67.2)
General practitioner	163 (54.5)
Obstetrician	142 (47.5)
Site of antenatal care (*n* = 270)	
GP’s surgery	85 (31.5)
Private obstetrician’s office	24 (8.9)
Outpatient department of a hospital	142 (52.6)
Community-based clinic	19 (7.0)
Type of delivery (*n* = 297)	
Vaginal	197 (66.3)
Planned C-section	49 (16.5)
Unplanned C-section	51 (17.2)
Parity (n-291)	
Primipara	123 (42.3)
Multipara	168 (57.7)
Maternal health (*n* = 299)	
Chronic health problem	41 (13.7)
Complication during pregnancy	94 (31.4)
Medical problem since delivery	19 (6.4)

^a^Missing responses were excluded from the analysis; valid percentages are reported

^b^Percentage reported for antenatal care providers is > 100 as women were instructed to indicate all that applied

**Table 2:** QPCQ factor (subscale) minimums, maximums, and means and standard deviations (SD)

Subscale	Minimum	Maximum	Mean (SD)
Factor 1 – Information Sharing	1.89	5.00	4.27 (0.55)
Factor 2 – Anticipatory Guidance	2.00	5.00	3.84 (0.65)
Factor 3 – Sufficient Time	1.80	5.00	4.13 (0.64)
Factor 4 - Approachability	2.00	5.00	4.17 (0.73)
Factor 5 - Availability	1.80	5.00	4.10 (0.63)
Factor 6 – Support and Respect	1.25	5.00	4.21 (0.62)
Total QPCQ	2.20	5.00	4.11 (0.55)

**Table 3:** Items on each factor, corrected item-total subscale correlations, Cronbach’s alpha if item deleted, and factor loadings

Factor (Subscale) items	Corrected item-total subscale correlation	Cronbach’s alpha if item deleted from subscale	Factor loading^a^
Factor 1: Information Sharing (9 items) Cronbach’s alpha = .89			
I was given adequate information about antenatal tests and procedures	.70	.88	.720
I was always given honest answers to my questions	.63	.88	.680
Everyone involved in my antenatal care received the important information about me	.61	.89	.632
I was screened adequately for potential problems with my pregnancy	.61	.88	.633
The results of tests were explained to me in a way I could understand	.67	.88	.692
My antenatal care provider(s) gave straightforward answers to my questions	.75	.87	.795
My antenatal care provider(s) gave me enough information to make decisions for myself	.67	.88	.779
My antenatal care provider(s) kept my information confidential	.66	.88	.682
I fully understood the reasons for blood work and other tests my antenatal care provider(s) ordered for me	.61	.88	.605
Factor 2: Anticipatory Guidance (11 items) Cronbach’s alpha = .88			
My antenatal care provider(s) gave me options for my birth experience	.54	.87	.609
I was given enough information to meet my needs about breastfeeding	.51	.87	.518
My antenatal care provider(s) prepared me for my birth experience	.60	.87	.650
My antenatal care provider(s) spent time talking with me about my expectations for labor and delivery	.67	.86	.687
I was given enough information about the safety of moderate exercise during pregnancy	.61	.87	.566
I received adequate information about my diet during pregnancy	.59	.87	.538
My antenatal care provider(s) was interested in how my pregnancy was affecting my life	.57	.87	.609
I was linked to programs in the community that were helpful to me	.56	.87	.513
I received adequate information about alcohol use during pregnancy	.54	.87	.542
I was given adequate information about depression in pregnancy	.57	.87	.517
My antenatal care provider(s) took time to ask about things that were important to me	.70	.86	.856
Factor 3: Sufficient Time (5 items) Cronbach’s alpha = .83			
I had as much time with my antenatal care provider(s) as I needed	.61	.80	.649
My antenatal care provider(s) was rushed	.50	.86	.487
My antenatal care provider(s) always had time to answer my questions	.73	.77	.811
My antenatal care provider(s) made time for me to talk	.71	.77	.807
My antenatal care provider(s) took time to listen	.71	.77	.859
Factor 4: Approachability (4 items) Cronbach’s alpha = .74			
My antenatal care provider(s) was abrupt with me	.46	.73	.516
I was rushed during my antenatal care visits	.57	.66	.678
My antenatal care provider(s) made me feel like I was wasting their time	.54	.68	.651
I was afraid to ask my antenatal care provider(s) questions	.58	.66	.696
Factor 5: Availability (5 items) Cronbach’s alpha = .83			
I knew how to get in touch with my antenatal care provider(s)	.51	.83	.642
Someone in my antenatal care provider(s)’s office always returned my calls	.54	.83	.615
My antenatal care provider(s) was available when I had questions or concerns	.69	.79	.805
I could always reach someone in the office/clinic if I needed something	.71	.78	.694
I could reach my antenatal care provider(s) by phone when necessary	.74	.77	.621
Factor 6: Support and Respect (12 items) Cronbach’s alpha = .95			
My antenatal care provider(s) respected me	.75	.95	.754
My antenatal care provider(s) respected my knowledge and experience	.75	.95	.743
My decisions were respected by my antenatal care provider(s)	.83	.95	.838
My antenatal care provider(s) was patient	.72	.95	.748
I was supported by my antenatal care provider(s) in doing what I felt was right for me	.79	.95	.794
My antenatal care provider(s) supported me	.81	.95	.833
My antenatal care provider(s) paid close attention when I was speaking	.77	.95	.803
My concerns were taken seriously	.78	.95	.826
I was in control of the decisions being made about my antenatal care	.75	.95	.754
My antenatal care provider(s) supported my decisions	.84	.95	.843
I was at ease with my antenatal care provider(s)	.75	.95	.760
My values and beliefs were respected by my antenatal care provider(s)	.76	.95	.778

^a^ For all factor loadings *p* value is <0.001

A significant positive correlation between the QPCQ total score and the Satisfaction subscale score of the PESPC provided additional support for construct validity (Pearson *r* = 0.67). Convergent validity was demonstrated by a significant positive correlation (*r* = 0.50) between the Anticipatory Guidance subscale of the QPCQ and the Empowerment/Self-care subscale of the PIPC. These are relatively good correlations as it has been suggested that an *r* of 0.70 is high for most psychosocial variables and that such correlations tend to be in the range of 0.20 to 0.40 [36]. As noted by Polit and Beck [36], “An instrument’s validity is not proved, established, or verified but rather is supported to a greater or lesser extent by evidence" (p. 342). The overall QPCQ had acceptable internal consistency reliability (Cronbach’s alpha = 0.97) as did each of the factors (subscales) (Cronbach’s alpha = 0.74 to 0.95) (see Table 3). The CFA analysis showed a reasonable fit of the pre-specified model to the dataset for the QPCQ in Australia (RMSEA = 0.061; 90 % CI, 0.058 – 0.065, CFI = 0.884, chi-square = 2022.63, *p* < 0.001, df = 995). In addition, CFA showed that all six factors in the questionnaire are correlated with each other, hence yielding an oblique factor structure for the QPCQ.

## Discussion

The 46-item QPCQ and each of the six subscales were validated through CFA in an Australian population. Validity was further confirmed through hypothesis-testing and assessment of convergent validity. The correlation between the QPCQ total score and the Satisfaction subscale score of the PESPC was lower in the Australian group of women compared to the group of Canadian women (Pearson *r* = 0.67 versus 0.81) as reported by Heaman et al. [28]. Also, the convergent validity of the QPCQ was not as robust when tested in an Australian population. In the initial testing in a Canadian population, there was a correlation between the Support and Respect subscale of the QCPQ and the Respectfulness/Emotional Support subscale of the PIPC as well as a correlation between the Anticipatory Guidance subscale of the QPCQ and the Empowerment/Self-care subscale of the PIPC [28]. The reasons for the differences in correlations in validity testing between the Australian and Canadian settings are not fully understood, but might be related to differences in participant characteristics. Women in the Australian sample started prenatal care at a later mean gestational age than women in the Canadian sample (12.9 vs 10.6 weeks); the Australian sample had a higher proportion of primiparous women (42.3 % vs 37.2 %) and women who received midwifery care (67.2 % vs 9.2 %), and a lower proportion of women who were married (58.1 % vs 67.3 %), born in the host country (65.6 % vs 75.4 %), and had less than a high school education (14.3 % vs 8.0 %) compared to women in the Canadian sample [28].

The questionnaire demonstrated acceptable internal consistency reliability when tested in an Australian population. The Cronbach’s alpha for the overall QPCQ of 0.97 was acceptable and similar to that determined in the Canadian population (0.96) as was the Cronbach’s alpha for each subscale in the Australian sample (0.74 to 0.95) compared to those in the Canadian sample (0.73 to 0.93) [28]. The test-retest reliability and temporal stability QPCQ were previously established [28].

The QPCQ is the first published instrument to comprehensively measure quality of antenatal care. The PIPC instrument developed by Wong et al. [13] measures only one dimension of quality. More recently, Barry et al. [37] developed the Patient-Provider Relationship Scale that similarly focuses on a single dimension and was developed specifically for use in a limited-resource setting. Beeckman et al. [38] developed the Content and Timing of Care in Pregnancy tool to assess whether women received recommended content based on clinical evidence and national and international antenatal care guidelines. The first iteration of the tool measures timing of the initiation of care and only three elements of content of care and the appropriateness of their timing: blood pressure assessment, blood tests, and ultrasound scans. The authors note that future work will incorporate additional elements of antenatal care.

The QPCQ enables researchers to assess relationships between quality of care and a variety of maternal and child outcomes, health-related behaviours, and use of other health services. It can be used to examine quality of antenatal care across regions, populations, and service delivery models, with subscale scores providing information about specific components of care. The QPCQ is intended to be completed by women after 36 weeks of pregnancy or within the first 6 weeks postpartum and was purposefully developed to be applicable to all women receiving antenatal care; it does not address quality of care specific to risk conditions [28]. A score can be calculated for the total QPCQ as well as for each of the subscales [28].

The QPCQ will also be useful in quality assurance and improvement initiatives designed to assess and improve the organization and processes of antenatal care, and ultimately to positively impact health-related outcomes. Elements of a high quality health system are effectiveness, patient-centredness, timeliness, efficiency, equity, safety, and “the seventh element of quality”, the care provider-patient relationship [1, 39]. Many of these elements are reflected in the QPCQ items. They incorporate the notions of effective, evidence-based practice (e.g., “I was given enough information about the safety of moderate exercise during pregnancy”), patient-centredness (e.g., “My antenatal care provider(s) was interested in how my pregnancy was affecting my life”), timeliness (e.g., “My antenatal care provider(s) was available when I had questions or concerns”), equity (e.g., “My values and beliefs were respected by my antenatal care provider(s)”), and the care provider-patient relationship (e.g., “My concerns were taken seriously”). The QPCQ items similarly reflect components of a recently introduced framework for quality maternal and newborn care, which emphasizes good quality clinical care, communication, education, information, and respect [7].

A strength of the study is the use of two sites in two states to provide a cross-section of women in Australia who use antenatal care services. Monash Health provides care for more than 13 % of women giving birth in public maternity services in Victoria [40] and they come from socio-economically diverse communities that are representative of the childbearing population. Wollongong Hospital also caters to a socio-economically diverse population that is representative of childbearing women in non-metropolitan Australia, although the proportions of women in the state is lower than in Victoria. The methodology was rigorous and was guided by the methodological framework for developing and testing measurement scales described by Streiner and Norman [32] that is widely used. The use of a convenience sample is a study limitation. The relatively high mean subscale scores suggest a potential selection bias in that women who received high quality care might have been more willing to participate in the study than those who received low quality care. The scores also might be a reflection of the fact that 67 % of study participants reported they saw a midwife; a previous study found that having a midwife as a care provider was the strongest predictor of high quality prenatal care [41]. The QPCQ requires testing in a variety of health care systems, service delivery models, and populations that are substantively different from the Canadian and Australian contexts to further substantiate its validity and reliability in diverse settings.

## Conclusion

The QPCQ is a valid and reliable self-report measure of overall quality of antenatal care and quality of care related to each of the subscales. It demonstrated acceptable internal consistency reliability and the six factors (subscales) were confirmed in an Australian population. This instrument can be used in research and in quality assurance and improvement initiatives. It fills a much needed gap in the scientific foundation for assessment of antenatal care practices and the benefits of quality antenatal care, and the QPCQ items reflect the Institute of Medicine’s [1] premise that “Good quality means providing patients with appropriate services in a technically competent manner, with good communication, shared decision making, and cultural sensitivity” (p. 232).
